# Machine Learning-Based Identification of Immune Inflammation-Related Genes as Shared Potential Diagnostic Biomarkers in Autism Spectrum Disorder and Atopic Dermatitis

**DOI:** 10.3390/biomedicines14051090

**Published:** 2026-05-12

**Authors:** Ruiling Yang, Fushen Zhang, Jufang Huang

**Affiliations:** Department of Anatomy and Neurobiology, Xiangya School of Basic Medical Sciences, Central South University, Changsha 410013, China

**Keywords:** autism spectrum disorder, atopic dermatitis, machine learning, bioinformatics

## Abstract

**Background**: ASD is a class of neurodevelopmental disorders with onset in early childhood, whereas AD is a common chronic inflammatory skin disease. An increasing number of studies suggest that immune dysregulation and inflammatory responses play important roles in the onset and progression of both conditions; however, their shared molecular mechanisms remain unclear. **Methods**: First, ASD-related and AD-related datasets were obtained from the GEO database. After removal of batch effects, the common DEGs between the two diseases were identified. Subsequently, 107 machine learning-based model configurations were employed to screen for key genes. Functional enrichment analyses and PPI network construction were performed to systematically explore their potential functions. Finally, the CIBERSORT was applied to analyze immune cell infiltration and to assess the correlation between hub gene expression and immune cell infiltration. **Results**: 164 common genes between ASD and AD were identified. GO and KEGG enrichment analyses revealed that these shared differentially expressed genes were mainly enriched in pathways related to immune regulation and inflammatory responses, suggesting that immuno-inflammatory processes may constitute an important biological basis linking ASD and AD. Further screening and validation using machine learning identified *BEX4*, *BIN2*, *BNIP3L*, *CCNO*, *JAK2*, *SLC39A7*, and *WASF3* as hub genes serving as common potential biomarkers for both diseases. Among them, *BIN2*, *SLC39A7*, and *JAK2* may represent key shared genes and demonstrated good diagnostic value in ROC curve and nomogram analyses. In addition, immune infiltration analysis indicated that these key genes were significantly correlated with the infiltration levels of multiple immune cell types, further supporting their potential roles in immune regulation. **Conclusions**: This study reveals potential shared immuno-inflammatory molecular mechanisms between ASD and AD. Genes screened based on 107 machine learning models were verified as potential diagnostic biomarkers for both diseases after integrated analysis, providing a theoretical basis for further investigation of their immune-related pathogenesis and early clinical diagnosis.

## 1. Introduction

Autism spectrum disorders are a class of neurodevelopmental disorders that occur in early childhood, characterized by deficits in social communication, restricted interests, and repetitive, stereotyped behaviors [1]. Globally, the prevalence of ASD is approximately 1% [2], while the average prevalence in Asia is 1.48 per 1000 individuals [3]. At present, the etiology of ASD remains unclear and is thought to involve both genetic and environmental factors, with heritability estimates ranging from 64% to 91% [4]. Converging hypotheses suggest that neuroinflammation, or at least interactions between the immune and nervous systems, may contribute to the pathogenesis of ASD in certain cases or populations [5].

Atopic dermatitis is a common chronic inflammatory skin disease characterized by pruritus and recurrent eczematous lesions, affecting more than 20% of children in high-income countries [5]. The number of cases has continued to rise over recent decades, and its chronic relapsing course, economic burden, and the need for family involvement in treatment markedly reduce the quality of life of patients and their families.

Studies have shown that the incidence of AD in patients with ASD is significantly higher than that in control populations, suggesting a comorbid tendency between the two conditions. For example, a systematic review and meta-analysis reported that patients with AD have a significantly elevated risk of ASD (pooled odds ratio ≈ 1.87), while individuals with ASD are more likely to develop AD than non-ASD controls, indicating a statistical association between ASD and AD [6].

Some studies suggest that ASD and AD share similar pathological mechanisms, including immune and genetic abnormalities [7]. As a multifactorial disorder, ASD is influenced not only by the interaction between genetic factors and inflammation, but also by oxidative stress during critical developmental periods, such as maternal immune activation [8]. Notably, these early-life immune and metabolic disturbances are also considered to be closely associated with the development of AD. For example, a recent study published in Nature reported that fluctuations in maternal glucocorticoids during pregnancy can induce fetal immune dysregulation, thereby increasing the risk of eczema in offspring after birth [9]. These findings suggest that ASD and AD may share a common etiological basis involving early immune abnormalities and inflammatory imbalance, which may contribute to their clinical comorbidity or overlapping risk. Therefore, ASD and AD may have closely related or similar pathogenic mechanisms; however, the precise underlying links remain unclear.

In this study, we employed bioinformatics approaches to explore the genetic interactions and potential pathogenic mechanisms underlying ASD and AD. First, gene expression data from the GEO database were used to identify common differentially expressed genes (DEGs) between ASD and AD. Subsequently, 107 machine learning-based model configurations were applied to screen for shared key genes (hub genes). Further bioinformatics analyses, including Gene Ontology (GO) functional annotation, Kyoto Encyclopedia of Genes and Genomes (KEGG) pathway enrichment, and protein–protein interaction (PPI) network construction, revealed a high degree of association between ASD and AD across multiple core pathways. Based on these analyses, we identified shared gene signatures between the two diseases and, combined with immune infiltration analysis and the assessment of correlations between hub genes and immune cells, provided new insights and a theoretical basis for elucidating the biological mechanisms linking ASD and AD.

## 2. Materials and Methods

**Figure 1:**
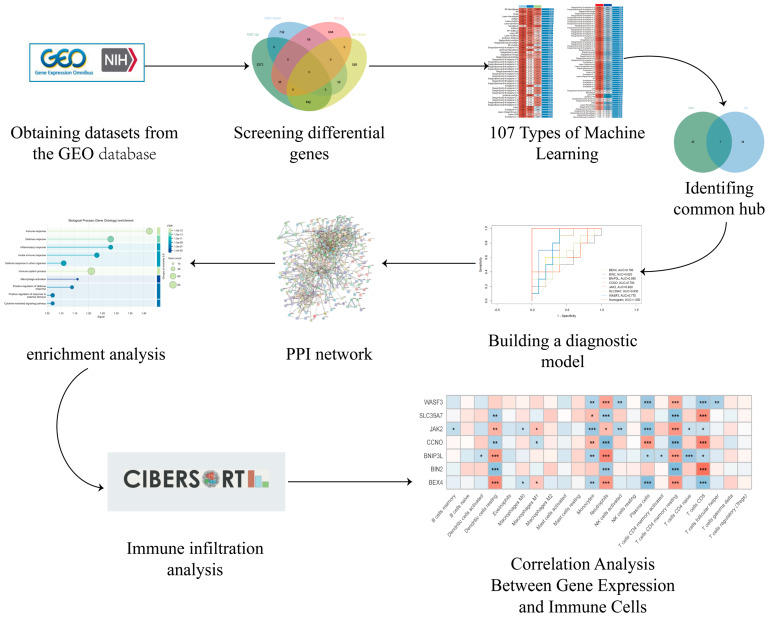
Analytical pipeline of this study. * *p* < 0.05, ** *p* < 0.01 and *** *p* < 0.001.

The study workflow is shown in Figure 1.

### 2.1. Data Collection and Preprocessing

ASD-related and AD-related datasets were retrieved from the Gene Expression Omnibus (GEO) database (http://www.ncbi.nlm.nih.gov/geo/ (accessed on 20 February 2026)). For ASD, the keywords “autism spectrum disorder” or “ASD” were used, with “human” as the organism filter. Two datasets, GSE18123 and GSE25507, were selected as ASD-related datasets (66 ASD patients vs. 33 healthy controls and 64 ASD patients vs. 82 healthy controls, respectively). GSE18123 was used as the training set, and GSE25507 as the testing set. Similarly, AD-related datasets GSE36842, GSE16161, GSE32924, and GSE6012 were retrieved. After batch effect correction, GSE36842 and GSE16161 were combined to form the training set, while GSE32924 and GSE6012 served as testing sets. After merging GSE36842 and GSE16161, we performed principal component analysis (PCA) to evaluate the presence of batch effects and the effect of correction. To quantitatively evaluate batch effects and the effectiveness of ComBat normalization, variance partitioning analysis was performed to estimate the contribution of batch factors to the total variance in the dataset, thereby assessing the extent of batch effect reduction before and after correction. In addition, the average silhouette score between samples was calculated to evaluate the stability of the clustering structure before and after batch correction, in order to determine whether the normalization process affected the underlying biological signals. The results are shown in Figure S1 of the Supplementary Materials. For each dataset, gene annotation information from the corresponding GPL platform was used, and when multiple probes mapped to the same gene, the probe with the highest expression value was retained. Gene expression matrices were then log_2_-transformed, and the “normalizeBetweenArrays” function from the R package limma was applied for normalization. Finally, normalized datasets with gene symbols were obtained.

### 2.2. Differential Gene Screening

After preprocessing each dataset, DEGs between disease and control groups for ASD and AD were calculated using the limma package in R. For ASD datasets, the thresholds were set as *p* < 0.05 and |fold change (FC)| > 1.2, whereas for AD datasets, the thresholds were *p* < 0.05 and |FC| > 2. In this study, FC refers to log_2_ fold change (log_2_FC), which represents the log-transformed fold change in expression levels between two groups.

A more stringent fold-change threshold (|FC| > 2) was applied to the AD datasets, mainly as a trade-off based on their data characteristics. Specifically, the AD data were integrated from multiple independent datasets. Although batch effect correction was performed, cross-platform and cross-batch integration may still introduce additional technical variation. Meanwhile, the sample size of this dataset was relatively small. These factors may increase the risk of false-positive results. Therefore, we adopted a stricter FC threshold to prioritize more robust and reproducible differential expression signals, thereby improving the specificity and reliability of the results.

In contrast, the ASD dataset had a larger sample size, a more consistent data source, and lower overall variability. A relatively relaxed threshold (|FC| > 1.2) was thus used to better capture subtle expression changes that may be biologically meaningful. Finally, the expression patterns of DEGs were visualized using the pheatmap and ggplot2 packages in R, generating heatmaps and volcano plots, respectively.

### 2.3. PPI Network and Enrichment Analysis

The shared DEGs between ASD and AD were imported into the STRING database (https://string-db.org/ (accessed on 28 February 2026)) [10] to obtain information on PPI networks. The organism was set to *Homo sapiens*, and the minimum required interaction score was set to “medium confidence” [11]. GO analysis, encompassing cellular component (CC), molecular function (MF), and biological process (BP) categories, was performed to characterize the gene sets and reveal their biological significance [12]. KEGG, a comprehensive database integrating genomic and chemical information, provides pathway maps to help interpret gene functions [13,14] KEGG enrichment analysis was used to identify the metabolic and signaling pathways in which the gene sets are involved.

### 2.4. Development and Evaluation of a Machine Learning-Based Diagnostic Model

Consistent with previous studies [15], a series of 12 commonly used machine learning algorithms were applied to develop a proficient classification prediction model to select potential biomarkers. These algorithms included Lasso, Ridge, Elastic Net (Enet), Stepwise GLM (Stepglm), Support Vector Machine (SVM), glmBoost, Linear Discriminant Analysis (LDA), Partial Least Squares Regression for GLM (plsRglm), Random Forest, Gradient Boosting Machine (GBM), XGBoost, and Naive Bayes. Integrated ASD and AD sample datasets were used, and model predictions were calculated using an ensemble approach. For model training, GSE18123, GSE36842, and GSE16161 were selected as training datasets, while batch effects in the testing datasets (GSE25507, GSE32924, and GSE6012) were corrected. Comprehensive ensemble validation techniques were applied to evaluate model performance. A systematic classification predictive model was developed by integrating 12 commonly used machine learning algorithms. The relevant machine learning model combinations and their performance are described in the Supplementary Materials (Supplementary Data S1–S5). Model performance was assessed by calculating the area under the receiver operating characteristic curve (AUC) for each model and for individual model genes, with results visualized using heatmaps. The performance of the optimal model was further evaluated using calibration curves and decision curve analysis (DCA).

### 2.5. Validation of Candidate Diagnostic Genes in Testing Cohorts

The GSE25507 dataset was used as the ASD testing cohort, while GSE32924 and GSE6012 served as the AD testing cohorts. A nomogram was constructed incorporating seven candidate genes with the help of the RMS package. Calibration curves were used to evaluate the accuracy of the nomogram, and DCA was further performed to assess its clinical utility.

### 2.6. Immune Cell Infiltration and Correlation Analysis

CIBERSORT analysis was performed in R for each sample in the ASD and AD training cohorts to determine the levels of immune cell infiltration. The LM22 file, containing 22 annotated gene signatures, was downloaded from the CIBERSORT website (http://cibersort.stanford.edu/ (accessed on 28 February 2026)). Using CIBERSORT with 1000 permutations, the proportions of 22 immune cell types were quantified based on the LM22 gene signatures. Results were visualized in R using the corrplot, colors, and ggplot2 packages. In addition, Spearman’s nonparametric correlation test was used to assess the relationships between immune cell infiltration levels and the hub genes identified through the above methods.

## 3. Results

### 3.1. Screening of DEGs in ASD and AD

**Figure 2:**
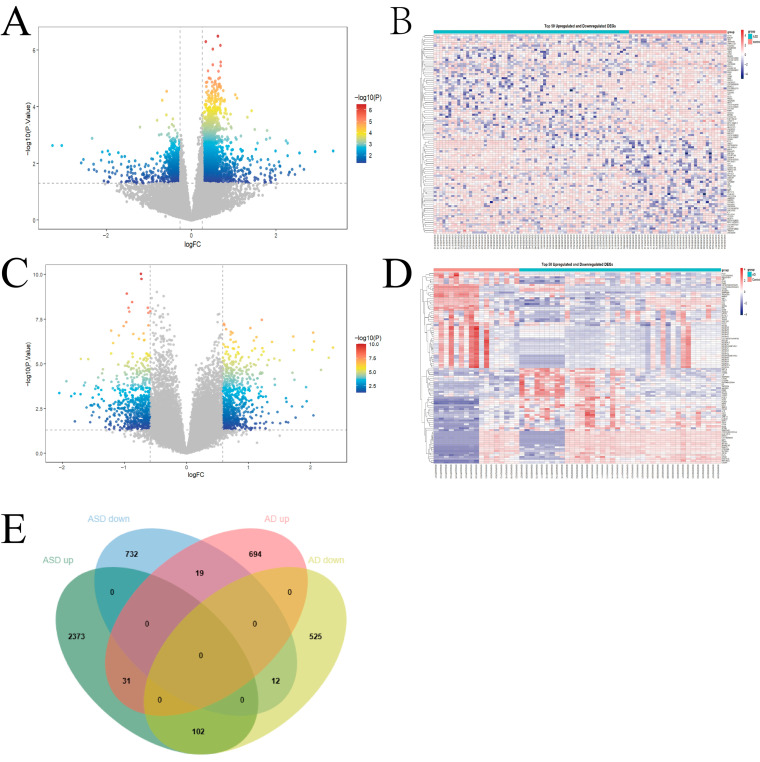
Differential Gene Expression Analysis in ASD and AD Datasets. (**A**). Volcano plot of DEGs in ASD blood samples. (**B**). Heatmap of the top 50 differentially expressed genes in ASD blood samples. (**C**). Volcano plot of DEGs in AD blood samples. (**D**). Heatmap of the top 50 differentially expressed genes in AD samples. (**E**). A total of 164 common DEGs were identified in both ASD and AD datasets.

Based on GSE18123, a total of 3296 DEGs were identified between ASD patients and healthy controls, including 2506 upregulated and 763 downregulated genes (Figure 2A,B). In addition, using GSE16161 and GSE36842, 1383 DEGs were identified between AD patients and healthy controls, comprising 744 upregulated and 639 downregulated genes (Figure 2C,D). Finally, 164 common DEGs shared between ASD and AD were identified, including 32 genes that were commonly upregulated and 12 genes that were commonly downregulated (Figure 2E).

### 3.2. Identification of Hub Genes in ASD Using Machine Learning Approaches

**Figure 3:**
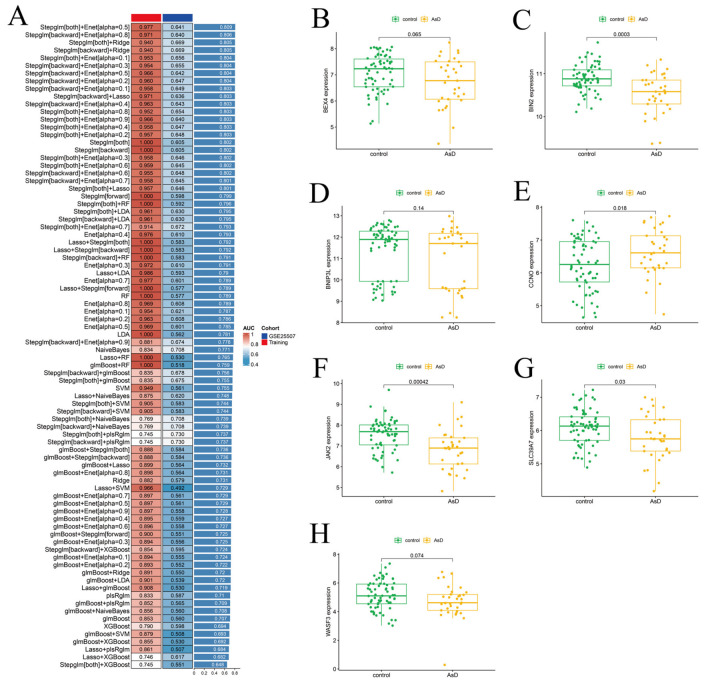
Construction and Evaluation of Risk Scores in the ASD Cohort. (**A**). AUC values of 107 machine learning algorithm combinations in the ASD cohort. (**B**). Expression level of BEX4 in ASD and healthy control groups. (**C**). Expression level of *BIN2* in the ASD training set. (**D**). Expression level of *BNIP3L* in the ASD training set. (**E**). Expression level of *CCNO* in the ASD training set. (**F**). Expression level of *JAK2* in the ASD training set. (**G**). Expression level of *SLC39A7* in the ASD training set. (**H**). Expression level of *WASF3* in the ASD training set.

Screening of Shared Hub Genes in ASD and AD Using 107 Machine Learning-based model configurations. A total of 107 machine learning-based model configurations were applied to the 164 common DEGs to further identify shared hub genes between ASD and AD. For ASD, GSE18123 was used as the training set and GSE25507 as the testing set. Among the 107 algorithms, Stepglm[both] + Enet[alpha = 0.5] showed the best performance (Figure 3A) and was therefore selected as the final algorithm for ASD. Using this approach, 32 hub genes were identified for subsequent analyses.

### 3.3. Identification of Hub Genes in AD Using Machine Learning Approaches

**Figure 4:**
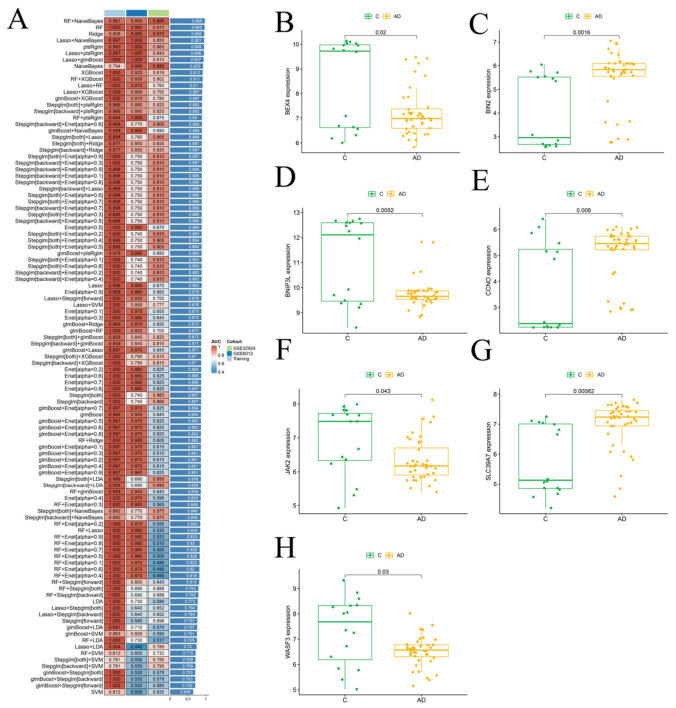
Construction and Evaluation of Risk Scores in the AD Cohort. (**A**). AUC values of 107 machine learning algorithm combinations in the AD cohort. (**B**). Expression level of *BEX4* in AD and healthy control groups. (**C**). Expression level of *BIN2* in the AD training set. (**D**). Expression level of *BNIP3L* in the AD training set. (**E**). Expression level of *CCNO* in the AD training set. (**F**). Expression level of *JAK2* in the AD training set. (**G**). Expression level of *SLC39A7* in the AD training set. (**H**). Expression level of *WASF3* in the AD training set.

In the AD cohort, GSE16161 and GSE36842 were merged, normalized, and standardized to serve as the training set, while GSE32924 and GSE6012 were used as testing sets. Using the same approach, the combination of RF + Naive Bayes was identified as the optimal algorithm (Figure 4A). This algorithm was then applied to screen hub genes in AD, resulting in a total of 47 hub genes. The hub genes identified in ASD and AD were then intersected, revealing seven overlapping genes: BEX4, BIN2, BNIP3L, CCNO, JAK2, SLC39A7, and WASF3. The expression levels of these seven genes were subsequently examined in the ASD training set (Figure 3B–H) and the AD training set (Figure 4B–H). This suggests that they may be involved in the inflammatory and neuroimmunomodulatory processes underlying the association between atopic dermatitis (AD) and autism spectrum disorder (ASD). The expression profiles of these genes in the AD validation set (GSE6012 and GSE32924) are shown in Figures S3 and S4 of the Supplementary Materials.

### 3.4. Validation of Hub Genes

**Figure 5:**
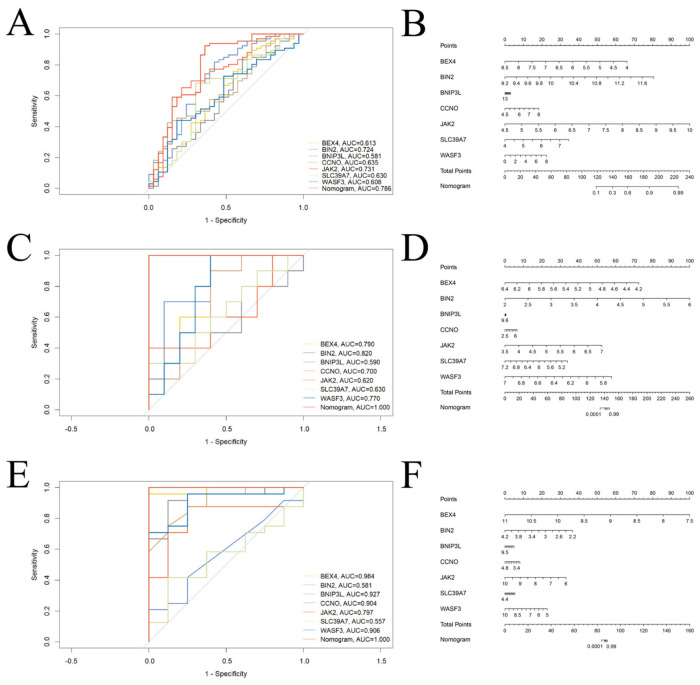
Validation of Candidate Diagnostic Genes. (**A**). Nomogram of candidate diagnostic genes in the GSE25507 dataset. (**B**). ROC curves of candidate diagnostic genes in the GSE25507 dataset. (**C**). Nomogram of candidate diagnostic genes in the GSE6012 dataset. (**D**). ROC curves of candidate diagnostic genes in the GSE6012 dataset. (**E**). Nomogram of candidate diagnostic genes in the GSE32924 dataset. (**F**). ROC curves of candidate diagnostic genes in the GSE32924 dataset.

To evaluate the diagnostic value of the shared hub genes identified from ASD and AD datasets, seven genes—*BEX4*, *BIN2*, *BNIP3L*, *CCNO*, *JAK2*, *SLC39A7*, and *WASF3*—were used to construct diagnostic models. The ASD dataset GSE25507 and the AD datasets GSE32924 and GSE6012 were used as testing cohorts to generate ROC curves and nomograms (Figure 5).

In the ASD testing set, ROC curve analysis indicated that *JAK2* and *BIN2* exhibited relatively high diagnostic performance, with AUC values of 0.731 and 0.724, respectively (Figure 5A). Meanwhile, *CCNO*, *SLC39A7*, and *BEX4* showed moderate diagnostic value in ASD, with AUCs of 0.635, 0.630, and 0.613, respectively (Figure 5A). The expression of *JAK2* corresponded to higher risk scores (points), suggesting that individuals with elevated *JAK2* and *BIN2* expression have a higher risk of ASD (Figure 5B). In the AD testing sets GSE6012 and GSE32924, ROC curve analysis demonstrated that *BEX4*, *BIN2*, *CCNO*, and *WASF3* had good diagnostic performance, while *BNIP3L* and *JAK2* also showed favorable diagnostic ability. Nomogram analysis further indicated that high expression of *BEX4* and *BIN2* was associated with higher risk scores, suggesting a close relationship between their elevated expression and increased AD risk (Figure 5C–F).

Notably, in both ASD and AD testing cohorts, the integrated diagnostic model (nomogram) based on these seven genes achieved AUC values of 0.786 and 1.0, respectively, demonstrating strong discriminative ability. An AUC value of 1 is clinically almost unattainable. We therefore performed 10-fold cross-validation again for these potential biomarkers in the AD validation set (Figure S3 in the Supplementary Materials). The results showed consistent model performance across different data splits (all C-index = 1.0). In addition, we conducted bootstrap resampling analysis, which revealed that model performance was mainly distributed in a high range, suggesting that the model has reasonable stability. Overall, these results indicate that the diagnostic model constructed from the seven hub genes identified through machine learning exhibits good diagnostic performance and potential clinical utility for both ASD and AD.

### 3.5. PPI Network and Functional Enrichment Analysis

**Figure 6:**
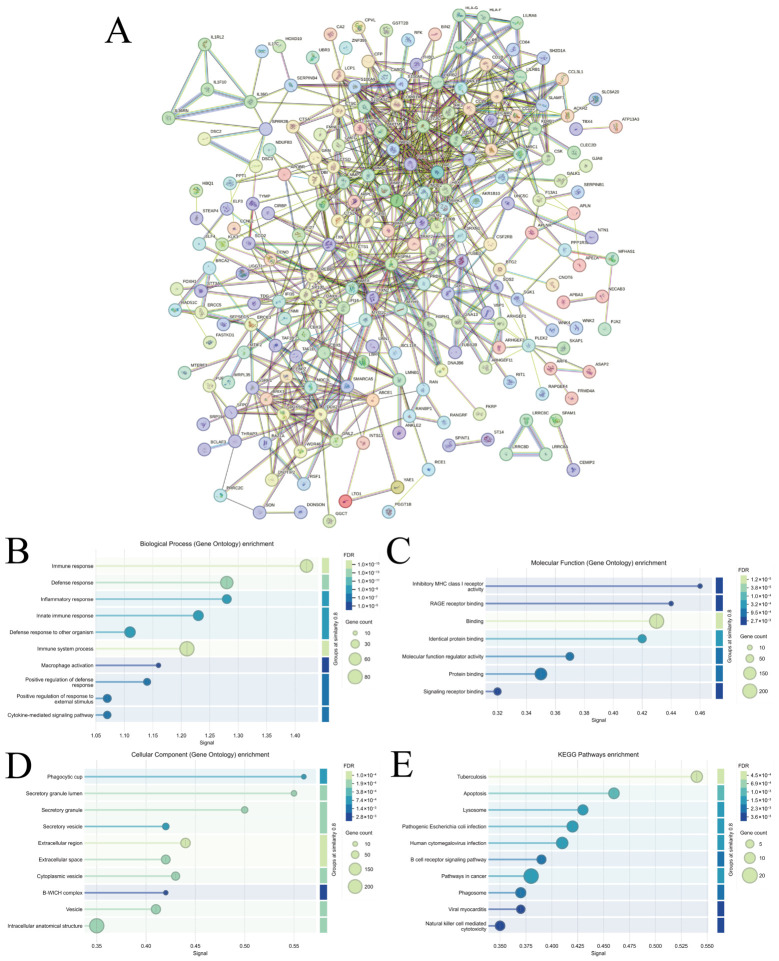
PPI Network and Functional Enrichment Analysis. (**A**). The PPI network from STRING. (**B**). The BP analysis. (**C**). The MF analysis. (**D**). The CC analysis. (**E**). The KEGG pathways enrichment.

To investigate the potential regulatory mechanisms of shared DEGs in ASD and AD, a PPI network was constructed using STRING (Figure 6A). The network consisted of 249 nodes (proteins) and 655 edges (interactions), with an average node degree of 5.26 (*p* < 0.005). Key proteins, including BIN2, CCNO, and JAK2, had multiple direct interacting partners.

Functional enrichment analysis (Figure 6B–E) revealed that, at the BP analysis, these proteins were significantly enriched in “immune response,” “defense response,” and “inflammatory response” (*p* < 0.005). Notably, CCNO was indirectly connected to several proteins involved in immune and inflammatory responses, such as RIPK3 and AIF1, which play critical roles in neurodegenerative and inflammatory diseases. In addition, at the MF analysis, enrichment was observed in inhibitory MHC class I receptor activity, RAGE receptor binding, and general binding. These results suggest that this protein cluster may regulate immune cell cytotoxicity through inhibitory immune receptor activity while simultaneously participating in inflammatory signal sensing and transduction via RAGE binding, indicating a dual role in the immune microenvironment of ASD and AD. Then, at the CC analysis, the proteins were mainly enriched in the phagocytic cup and secretory granule lumen. BIN2, a membrane-curvature sensing protein containing an N-BAR domain, is known to localize to the phagocytic cup in macrophages and regulate actin cytoskeleton remodeling during phagocytosis. These subcellular localizations suggest that BIN2 may exert dual regulatory functions in immune cell phagocytosis and secretion pathways, providing a structural basis for its involvement in innate immune responses. Analysis of KEGG pathways reveals that these proteins are enriched in the Tuberculosis, Apoptosis and Lysosome pathways. Although the Tuberculosis pathway is named after the disease, its core content actually constitutes a general model of the host immune response, encompassing key processes such as phagocytosis, lysosomal degradation, inflammatory signal transduction and antigen presentation; this aligns closely with phagosome localisation and MHC receptor function; The Lysosome pathway further supports their role in antigen processing and degradation; the Apoptosis pathway, meanwhile, suggests that these proteins may be involved in determining the fate of infected or stressed cells.

### 3.6. Immune Infiltration and Correlation Analysis in ASD

Based on the above PPI network and enrichment analyses, we found that the shared DEGs between ASD and AD—particularly the seven identified hub genes (e.g., *BIN2*, *CCNO*, and *JAK2*)—were significantly enriched in key biological processes such as immune response, inflammatory response, and defense response. These genes are widely involved in immune-related pathways, including phagocytosis, antigen processing and presentation, inflammatory signaling, and cell fate regulation. At both the molecular function and cellular component levels, these genes were closely associated with immune receptor activity, phagocytic structures, and secretory pathways, further suggesting their important roles in regulating the immune microenvironment. Collectively, these findings indicate that these hub genes may play critical regulatory roles in the immuno-inflammatory mechanisms underlying ASD and AD. Therefore, we further performed immune infiltration analysis to explore the interactions between these key genes and immune cells, aiming to better elucidate the shared immune regulatory mechanisms of the two diseases.

CIBERSORT analysis was applied to the ASD cohort (GSE18123) and AD cohorts (GSE36842 and GSE16161) to estimate the relative abundance of 22 immune cell subtypes based on gene expression profiles.

**Figure 7:**
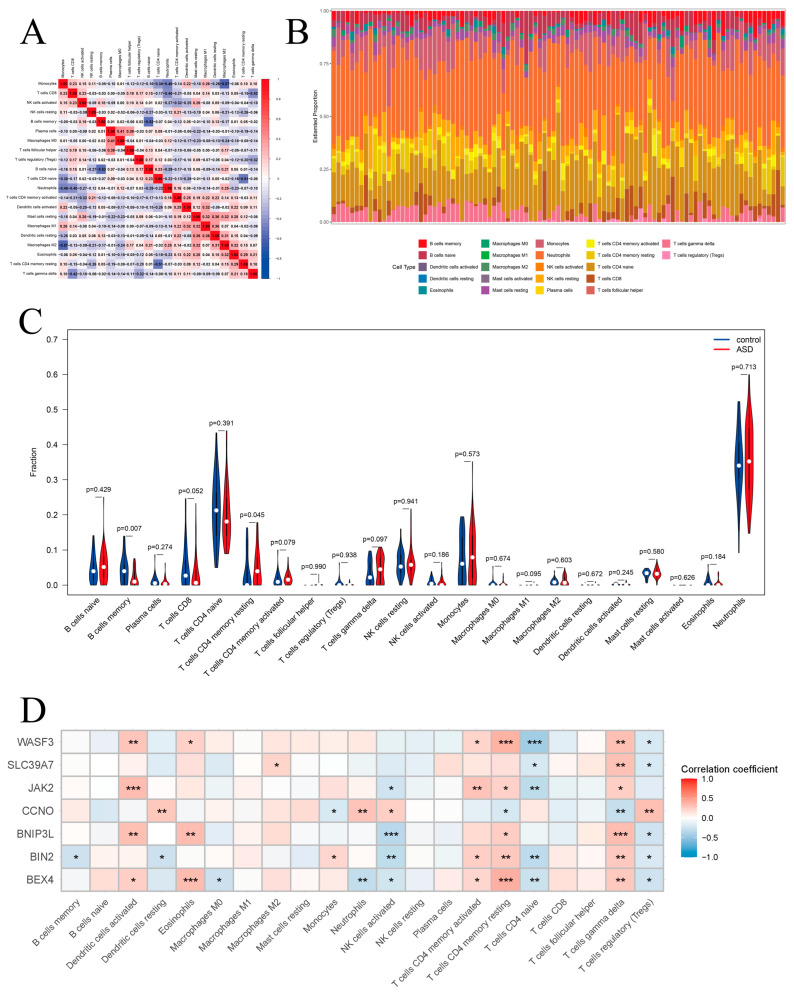
Immune Cell Infiltration in the ASD Cohort. (**A**). Correlation among immune cell types. (**B**). Proportions of immune cells across samples (stacked bar plot). (**C**). Violin plots showing differences in immune cell proportions between ASD and control groups. (**D**). Heatmap of correlations between immune cell infiltration and the seven potential biomarkers.* *p* < 0.05, ** *p* < 0.01 and *** *p* < 0.001.

In the ASD cohort, we first assessed the correlations among various immune cell types (Figure 7A). For example, resting mast cells were positively correlated with M1 macrophages, resting dendritic cells, and M2 macrophages; resting dendritic cells were positively correlated with both M1 and M2 macrophages; activated memory CD4^+^ T cells showed positive correlations with activated dendritic cells, M2 macrophages, and resting dendritic cells, whereas monocytes were negatively correlated with naïve CD4^+^ T cells, neutrophils, and M2 macrophages. The composition of 22 immune cell types in each sample was then visualized using bar plots (Figure 7B), where different colors represent the proportions of immune cells in each sample. The results indicated that neutrophils, CD4^+^ T cells, mast cells, memory B cells, regulatory T (Treg) cells, and NK cells were the predominant infiltrating immune cells in the ASD cohort. Furthermore, the Wilcoxon test was used to identify significant differences in immune cell infiltration between ASD patients and healthy controls. The results showed that two immune cell types exhibited significant differences (Figure 7C): memory B cell infiltration was significantly decreased (*p* < 0.005), whereas resting memory CD4^+^ T cell infiltration was significantly increased (*p* < 0.05). We also analyzed the correlations between hub genes and immune cell infiltration levels (Figure 7D). The results demonstrated that the seven potential biomarkers (*BEX4*, *BIN2*, *BNIP3L*, *CCNO*, *JAK2*, *SLC39A7*, and *WASF3*) were significantly associated with multiple immune cell types. For instance, the expression levels of *BEX4*, *BIN2*, *BNIP3L*, *JAK2*, and *WASF3* were positively correlated with resting memory CD4^+^ T cell infiltration (*p* < 0.05), whereas *CCNO* expression showed a negative correlation with resting memory CD4^+^ T cells (*p* < 0.005). In addition, *BIN2* expression was negatively correlated with memory B cell infiltration (*p* < 0.05).

In summary, the immune infiltration landscape in the ASD cohort was markedly altered, characterized by decreased memory B cells and increased resting memory CD4^+^ T cells, suggesting a potential imbalance between humoral and cellular immunity. Correlation analysis further indicated that most hub genes were closely associated with immune cell infiltration, supporting their roles in regulating the immune microenvironment. Notably, *BIN2*, *BEX4*, and *JAK2* exhibited consistent expression patterns in both ASD and AD, suggesting that they may play more critical roles in the shared pathogenesis of the two diseases. In ASD, *BEX4*, *BIN2*, and *JAK2* were positively correlated with activated dendritic cells and resting memory CD4^+^ T cell infiltration, while *BEX4* was negatively correlated with neutrophil infiltration and *BIN2* was negatively correlated with memory B cells. Therefore, these three genes were selected for focused analysis, and their significant associations with immune cell infiltration suggest that they may contribute to the immuno-inflammatory processes of ASD by regulating specific immune cell populations, such as memory CD4^+^ T cells and B cells.

### 3.7. Immune Infiltration and Correlation Analysis in AD

**Figure 8:**
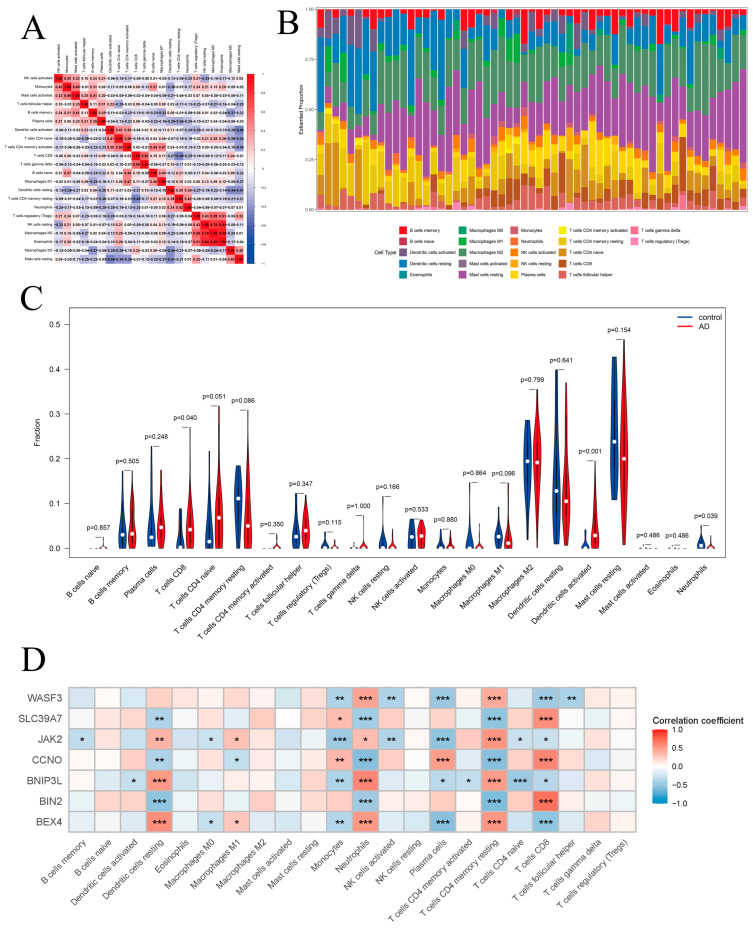
Immune Cell Infiltration in the AD Cohort. (**A**). Correlation among immune cell types. (**B**). Proportions of immune cells across samples (stacked bar plot). (**C**). Violin plots showing differences in immune cell proportions between AD and control groups. (**D**). Heatmap of correlations between immune cell infiltration and the seven potential biomarkers. * *p* < 0.05, ** *p* < 0.01 and *** *p* < 0.001.

In the AD cohort, we similarly evaluated the correlations among various immune cell types (Figure 8A). The results showed that resting NK cells were positively correlated with M0 macrophages, regulatory T (Treg) cells, and eosinophils; resting dendritic cells were positively correlated with both M1 and M2 macrophages; activated memory CD4^+^ T cells were positively correlated with M1 macrophages and naïve B cells; whereas mast cells were negatively correlated with dendritic cells, activated memory CD4^+^ T cells, and naïve CD4^+^ T cells. The proportions of 22 immune cell types in each sample were then visualized using bar plots (Figure 8B). The results indicated that resting dendritic cells, resting CD4^+^ T cells, resting mast cells, resting memory CD4^+^ T cells, M2 macrophages, and naïve CD4^+^ T cells were the predominant infiltrating immune cells in the AD cohort. Furthermore, the Wilcoxon test was applied to identify significant differences in immune cell infiltration between AD patients and healthy controls. The results showed that two immune cell types exhibited significant differences (Figure 8C): neutrophil infiltration was significantly decreased (*p* < 0.05), while CD8^+^ T cell infiltration was significantly increased (*p* < 0.05). Further correlation analysis revealed that hub genes are closely associated with the levels of infiltration of various immune cell types (Figure 8D), and that different genes exhibit distinct regulatory patterns. Specifically, *BEX4*, *BNIP3L*, *JAK2* and *WASF3* were positively correlated with resting memory CD4^+^ T cells, whereas *BIN2*, *CCNO* and *SLC39A7* were negatively correlated (*p* < 0.05), suggesting that these genes may have distinct functional roles in immune regulation.

Notably, *BIN2* and *JAK2* were negatively correlated with multiple immune cell types, including dendritic cells, neutrophils, and various T cell subsets (*p* < 0.05), indicating their potential roles in modulating immune cell infiltration and reshaping the immune microenvironment. In AD, *BEX4* was positively correlated with resting dendritic cells, neutrophils, and resting memory CD4^+^ T cells, but negatively correlated with CD8^+^ T cells. In contrast, *BIN2* was positively correlated with CD8^+^ T cells and negatively correlated with resting dendritic cells, neutrophils, and resting memory CD4^+^ T cells. Consistent with findings in ASD, *BIN2*, *BEX4*, and *JAK2* exhibited similar expression patterns across both diseases and were strongly associated with immune cell infiltration, further supporting their key roles in the shared immuno-inflammatory mechanisms underlying ASD and AD.

## 4. Discussion

ASD is a neurodevelopmental disorder with onset in early childhood, characterized by deficits in social communication, restricted interests, and repetitive stereotyped behaviors. Although its etiology remains unclear, it is generally believed to result from the combined effects of genetic and environmental factors. In recent years, accumulating evidence has suggested that neuroinflammation and interactions between the immune and nervous systems may play important roles in the pathogenesis of certain ASD cases [16]. Eczema, also known as AD, is a common chronic inflammatory skin disease characterized by pruritus and recurrent eczematous lesions [5]. Notably, AD is not only a common comorbidity in children with ASD, but individuals with AD also exhibit an increased risk of developing ASD [6]. Previous studies [17,18], have suggested that ASD and AD may share similar pathological mechanisms, such as immune dysregulation and genetic susceptibility, indicating the possibility of common or interconnected pathogenic pathways; however, the underlying biological basis remains to be further elucidated.

Using a combination of 107 machine learning-based model configurations and bioinformatics approaches, we identified seven shared potential biomarkers between ASD and AD: *BEX4*, *BIN2*, *BNIP3L*, *CCNO*, *JAK2*, *SLC39A7*, and *WASF3*. Among these, *BIN2* and *SLC39A7* were consistently upregulated in both ASD and AD, whereas *JAK2* was consistently downregulated in both conditions. Subsequent construction of the protein–protein interaction (PPI) network and pathway enrichment analysis revealed that the shared DEGs between ASD and AD were primarily enriched in immune regulation and inflammatory response pathways, suggesting that immuno-inflammatory processes may play a crucial role in their common pathogenesis. Based on these findings, we further performed immune infiltration analysis, which demonstrated that the identified potential biomarkers were significantly correlated with the infiltration levels of multiple differential immune cell types in both ASD and AD.

In the immune infiltration analysis, in ASD, resting memory CD4^+^ T cell infiltration was positively correlated with the expression of *BEX4*, *BIN2*, *BNIP3L*, *JAK2*, and *WASF3* (*p* < 0.05), but negatively correlated with *CCNO* expression (*p* < 0.005). In addition, memory B cell infiltration was negatively correlated with BIN2 expression (*p* < 0.05). In AD, CD8^+^ T cell infiltration was positively correlated with *BIN2*, *CCNO*, and *SLC39A7* expression (*p* < 0.05), but negatively correlated with *BEX4*, *BNIP3L*, *JAK2*, and *WASF3* expression (*p* < 0.05). Furthermore, neutrophil infiltration was positively correlated with *BEX4*, *BNIP3L*, *JAK2*, and *WASF3* expression (*p* < 0.05), while negatively correlated with *BIN2*, *CCNO*, and *SLC39A7* expression (*p* < 0.05) *BIN2* and *SLC39A7* were both upregulated in ASD and AD, suggesting that they may promote disease progression by regulating immune cell activation and inflammatory responses. In contrast, *JAK2* was downregulated in both conditions, indicating that it may contribute to alterations in the immune microenvironment by affecting immune-related signaling pathways, such as cytokine-mediated signal transduction. These findings suggest that *BIN2*, *SLC39A7*, and *JAK2* may be involved in key processes of immune cell infiltration, thereby contributing to the shared pathogenesis of ASD and AD. However, immune infiltration analysis was performed using the CIBERSORT algorithm, which estimates immune cell proportions based on bulk transcriptomic data. The LM22 signature matrix employed by CIBERSORT was originally derived from peripheral blood immune cells. Consequently, its application to skin tissue samples may introduce bias due to differences in tissue-specific gene expression profiles and immune cell activation states. Immune cells residing in tissues may exhibit distinct transcriptional characteristics from those in circulation, which could compromise the accuracy of the deconvolution results. Furthermore, CIBERSORT provides computational estimates rather than direct experimental measurements, and its outputs may be affected by sample heterogeneity and data processing pipelines. Therefore, the interpretation of immune infiltration results should be treated with caution. Further validation using experimental approaches or alternative computational methods is warranted. Given that the datasets used in this study (e.g., GSE36842) were primarily derived from skin tissue, future validation using skin biopsy samples would provide the most direct evidence for the identified biomarkers. In addition, considering the feasibility of obtaining peripheral blood in clinical practice, peripheral blood mononuclear cells (PBMCs) may serve as a practical alternative for validation, particularly for evaluating systemic immune-related biomarkers.

BIN2 (Bridging Integrator 2) is a BAR domain–containing protein highly expressed in immune cells, primarily involved in cytoskeletal remodeling and membrane dynamics, particularly in macrophage function, phagocytosis, and regulation of inflammatory responses [19,20]. Studies have shown that BIN2 can regulate immune cell chemotaxis and activation by modulating the actin cytoskeleton, thereby participating in inflammatory and immune responses [21]. SLC39A7 (Solute Carrier Family 39 Member 7), a member of the zinc transporter (ZIP) family, is mainly localized to the membranes of the endoplasmic reticulum and Golgi apparatus. It functions to transport zinc ions from intracellular organelles into the cytoplasm, thereby maintaining intracellular zinc homeostasis [22]. Zinc is an essential cofactor in immune regulation and signal transduction; thus, SLC39A7 plays an important role in immune cell development, inflammatory responses, and the regulation of cell proliferation and apoptosis [23]. This is consistent with our findings from the PPI network and enrichment analyses, which indicate that both BIN2 and SLC39A7 are involved in the regulation of immuno-inflammatory responses. Multiple studies have shown that [24,25,26], he levels of various inflammatory cytokines are elevated in the blood of ASD patients [26,27,28], and immune activation and inflammatory responses can exacerbate fetal brain inflammation, thereby affecting neurodevelopment and increasing the risk of ASD. Similarly, AD is a disease driven by immune dysregulation and inflammatory responses, in which cytokines such as IL-4, IL-13, and Th2-related factors play key roles in its pathogenesis [27]. Moreover, certain inflammatory mediators can further promote skin inflammation and impair barrier function, thereby exacerbating the progression of AD [28]. Therefore, we speculate that immuno-inflammatory responses may represent a key biological mechanism linking ASD and AD. Our immune infiltration analysis revealed that the identified potential biomarkers were significantly associated with the infiltration levels of multiple immune cell types, suggesting that these genes may contribute to the formation of disease-related inflammatory microenvironments by regulating immune cell activation or recruitment. Notably, *BIN2* and *SLC39A7* were both upregulated in the two diseases and correlated with the infiltration of multiple immune cell types, further supporting their potential roles in immuno-inflammatory regulation. In addition, we revealed that *JAK2* was downregulated in both ASD and AD. JAK2 (Janus Kinase 2) is a key member of the JAK-STAT signaling pathway and a non-receptor tyrosine kinase that plays a critical role in signal transduction for various cytokine receptors [29]. *JAK2* is involved in regulating immune cell differentiation, proliferation, and cytokine production, and it plays important roles in immuno-inflammatory responses and the development of multiple diseases, including autoimmune and inflammatory disorders [30,31]. Therefore, the downregulation of *JAK2* observed in this study may contribute to immune dysregulation by affecting JAK-STAT and related signaling pathways, thereby participating in the pathogenesis of ASD and AD. This suggests that reduced *JAK2* expression could disrupt immune homeostasis, influencing disease onset and progression in both conditions.

In the process of atopic dermatitis (AD)-associated pruritus and neuro-immune crosstalk, inflammatory cytokines mainly activate downstream transcriptional programs through the JAK2-mediated JAK-STAT signaling axis and amplify the inflammatory cascade in the local skin microenvironment. This signal not only regulates immune cell function, but also upregulates pruritus-related cytokines such as IL-4, IL-13, and IL-31 by promoting STAT-dependent transcription, thereby enhancing the expression and excitability of ion channels including TRPV1 and TRPA1 in sensory neurons, leading to sensitization of peripheral sensory neurons and amplification of pruritic signals. Therefore, inhibiting *JAK2* can block the neuroactivation process driven by inflammatory factors upstream of signal transmission, thereby alleviating the pruritic phenotype [32]. Furthermore, in the chronic inflammatory microenvironment of AD, imbalanced macrophage polarization is an important mechanism underlying persistent inflammation. Among them, M1 macrophages, upon stimulation with IFN-γ or LPS, are synergistically activated through JAK2/STAT1 and NF-κB signaling, continuously upregulating pro-inflammatory factors including TNF-α, IL-6, and IL-1β. In contrast, M2 macrophages are mainly induced via the STAT6 signaling axis and secrete IL-10 and TGF-β1 to participate in anti-inflammatory responses and tissue repair [33,34,35]. Thus, the JAK-STAT pathway acts as an upstream hub regulating the M1/M2 balance of macrophages and indirectly shapes the excitability threshold of the neural microenvironment by altering the local cytokine profile [36,37]. In addition, *SLC39A7* can affect the expression of inflammatory factors by regulating macrophage polarization, promoting the upregulation of pro-inflammatory mediators and shifting the M1/M2 balance [37]. From the perspective of neurodevelopmental disorders, the above-mentioned JAK-STAT axis and zinc homeostasis-related pathways also potentially overlap with the neuroimmune dysfunction hypothesis of autism spectrum disorder (ASD). On the one hand, JAK2/STAT signaling is thought to be involved in glial cell activation, synaptic pruning, and maintenance of neural circuit homeostasis during neurodevelopment, and its aberrant activation may lead to disrupted neuroimmune homeostasis [38,39,40]. On the other hand, dysregulated zinc homeostasis (involving transporters such as SLC39A7) has been proposed to affect synaptogenesis and the excitatory/inhibitory (E/I) balance, which is consistent with the E/I imbalance hypothesis observed in ASD [41,42]. Moreover, the regulation of neuroinflammation and synaptic plasticity associated with BIN2 suggests that it may exert a refined regulatory effect on neural networks during neural development by modulating immune signals, playing a potential role in the shared neuroimmune developmental axis of AD and ASD. Notably, BIN2 has also been reported to participate in neuroinflammation, neuronal fate determination, and processes related to the JAK-STAT/NF-κB signaling pathway, suggesting that it may coordinate with JAK2 in the inflammatory-neural crosstalk network to regulate immune responses and neural sensitization in AD [43].

In the subsequent ROC curve and nomogram analyses for both diseases, we found that *BIN2*, *SLC39A7*, and *JAK2* exhibited consistent differential expression patterns in ASD and AD, suggesting their potential as potential biomarkers capable of simultaneously indicating both disease states. Therefore, we propose that *BIN2*, *SLC39A7*, and *JAK2* represent important molecular markers for studying comorbidity or shared mechanisms between ASD and AD. These findings indicate that multi-gene integrative analyses not only enhance diagnostic capability for ASD and AD but also provide a foundation for developing more robust and reliable molecular diagnostic models, offering potential theoretical support for early clinical screening, risk assessment, and precise diagnosis.

In summary, this study combined machine learning-based model configurations with bioinformatics analyses to identify and validate shared potential biomarkers between ASD and AD and further elucidated their key roles in immune regulation and inflammation-related pathways. Immune infiltration analysis demonstrated that these hub genes were significantly associated with the infiltration levels of multiple immune cell types, suggesting that alterations in the immune microenvironment may play a critical role in the shared pathogenesis of ASD and AD. These findings provide a novel perspective for understanding the potential molecular connections between ASD and AD and lay a theoretical foundation for future investigations into potential diagnostic biomarkers and potential therapeutic targets.

### Limitations

However, this study still has certain limitations. First, the sample size used for model construction and validation in this study is relatively limited (training set *n* = 33, testing set *n* = 20), which may affect the stability and generalization ability of the model. Under small sample conditions, model performance is easily affected by data distribution, which may lead to overestimation of performance. Therefore, although the model developed in this study achieved perfect classification performance on the current dataset, this result may be subject to spectrum bias. The current case and control samples may exhibit a high degree of distinguishability based on molecular characteristics, which may, to some extent, overestimate the model’s performance. In a real-world clinical setting, patients typically present with multiple comorbidities, variations in disease stage, and testing noise; these factors may reduce the model’s actual predictive capability. Second, although an independent external dataset was used for validation in this study, further validation in larger-scale and more diverse population cohorts is still needed to confirm the robustness and clinical applicability of the screened biomarkers. Third, the machine learning methods in this study were mainly applied to feature screening of candidate genes and construction of prediction models, and the candidate genes were derived from preliminary differential expression analysis. Therefore, although this integrated analysis strategy helps improve the efficiency of biomarker screening, the relevant results should be interpreted in conjunction with the overall analysis process and should not be regarded as completely independent data-driven discoveries. Finally, all data used in this study were obtained from public databases (GEO), and there may be differences in experimental platforms, batch effects, and population characteristics between different datasets, all of which may affect the robustness of the results. In addition, the results of this study still lack experimental validation (such as qPCR) and clinical sample support. Therefore, the screened biomarkers still need further validation in independent clinical cohorts and experimental studies, and their actual clinical application value remains to be confirmed.

## 5. Conclusions

This study systematically explored the potential shared molecular mechanisms between ASD and AD by integrating bioinformatics analyses with machine learning approaches. Our results demonstrated significant overlap between the two diseases in immune regulation and inflammation-related pathways, suggesting that immune dysregulation may play a critical role in their common pathogenesis. Further analyses identified *BIN2*, *SLC39A7*, and *JAK2* as key shared genes between ASD and AD. These genes not only exhibited consistent expression patterns in both conditions but were also closely associated with the infiltration levels of multiple immune cell types and showed strong diagnostic potential in ROC curve and nomogram analyses. Therefore, *BIN2*, *SLC39A7*, and *JAK2* may serve as important molecular potential biomarkers linking ASD and AD and hold potential diagnostic value. This study provides new insights into the immune-related pathogenic mechanisms underlying ASD and AD and offers a theoretical foundation for future research on early diagnosis and targeted interventions for these diseases.

## Data Availability

The database described in the article is available upon reasonable request from the authors.

## Supplementary Materials

The following supporting information can be downloaded at: https://www.mdpi.com/article/10.3390/biomedicines14051090/s1. Figure S1 Effects of batch effect removal on AD datasets GSE36842 and GSE16161; Figure S2 Ten-fold cross-validation of the reported AUC values in the atopic dermatitis validation cohort; Figure S3 [44]. Expression levels of hub genes in the AD validation dataset GSE6012; Figure S4 Expression levels of hub genes in the AD validation dataset GSE32924; Supplementary Data S1 and S2 The performance of predictive models in ASD training and testing cohorts; Supplementary Data S3 Each algorithm model classifies each sample in the ASD cohort; Supplementary Data S4 Each algorithm model classifies each sample in the AD cohort; Supplementary Data S5 All the algorithm models used in this research study.

## Abbreviations

The following abbreviations are used in this manuscript:

AD	Atopic dermatitis
ASD	Autism spectrum disorders
AUC	Area under the receiver operating characteristic curve
BIN2	Bridging integrator 2
BP	biological process
CC	cellular component
DCA	decision curve analysis
DEG	differentially expressed gene
GBM	gradient boosting machine
GEO	gene expression omnibus
GO	gene ontology
JAK2	janus kinase 2
KEGG	kyoto encyclopedia of genes and genomes
LASSO	least absolute shrinkage and selection operator
LDA	linear discriminant analysis
MF	molecular function
PPI	protein–protein interaction
plsRglm	partial least squares regression for GLM
RF	random forest
SVM	support vector machine
ZIP	zinc transporter
