# Supplementary material for: Machine Learning-Based Identification of Immune Inflammation-Related Genes as Shared Potential Diagnostic Biomarkers in Autism Spectrum Disorder and Atopic Dermatitis
Source: Biomedicines. 2026 May 12;14(5):1090. doi: 10.3390/biomedicines14051090 (PMC13204341; doi:10.3390/biomedicines14051090)
Supplement: Supplementary file 1 [file biomedicines-14-01090-s001.zip › Supplementary file .pdf]

Supplementary materials

1. The Effect of Data Preprocessing on the AD Training Cohort

In this study, for the expression data from different datasets (GSE36842 and GSE16161), we first performed data preprocessing, including normalization using the normalizeBetweenArrays function in the limma package to reduce the distribution differences between samples. Subsequently, the ComBat algorithm in the sva package was used to correct potential batch effects, so as to improve the reliability of data integration.

Meanwhile, in accordance with the reviewers' suggestions, we added principal component analysis (PCA) to evaluate the existence of batch effects and the effect of correction. Before batch correction, the PCA results showed that the samples exhibited a certain degree of separation by dataset source (GSE16161 and GSE36842) along the first principal component (Dim1), indicating the presence of potential batch effects (Figure 1 A). Although the two groups of samples were not completely separated, there was a significant shift in their distribution centers, and there were differences in the degree of dispersion between different datasets. After applying the ComBat method for correction, the sample distributions of different datasets became significantly mixed, and the batch-related clustering phenomenon was significantly weakened, indicating that the batch effects had been effectively eliminated, thereby improving the reliability of data integration (Figure 1 B). In addition, after batch correction, the AD group and the control group still showed a clear separation trend (Figure 1 C), indicating that the biological differences were well preserved.

To quantitatively assess batch effects, we additionally performed an analysis of variance (Figure 1D) to quantify the contribution of batch effects to overall variation before and after ComBat correction. The results showed that the proportion of variance explained by the batch factor decreased from 0.645 before correction to 0.415 after correction, indicating that the ComBat processing significantly reduced the impact of batch effects on the data. To further assess whether the correction process interfered with underlying biological structures, we calculated the silhouette coefficient for the samples (Figure 1E). The results showed that this metric remained stable before and after correction (both 0.622), indicating that whilst batch effects were attenuated, the original clustering structure among samples remained largely unchanged. In summary, these quantitative analysis results are consistent with the observations in the PCA plot, further confirming that ComBat can effectively reduce technical batch effects whilst maintaining the integrity of the biological signal.

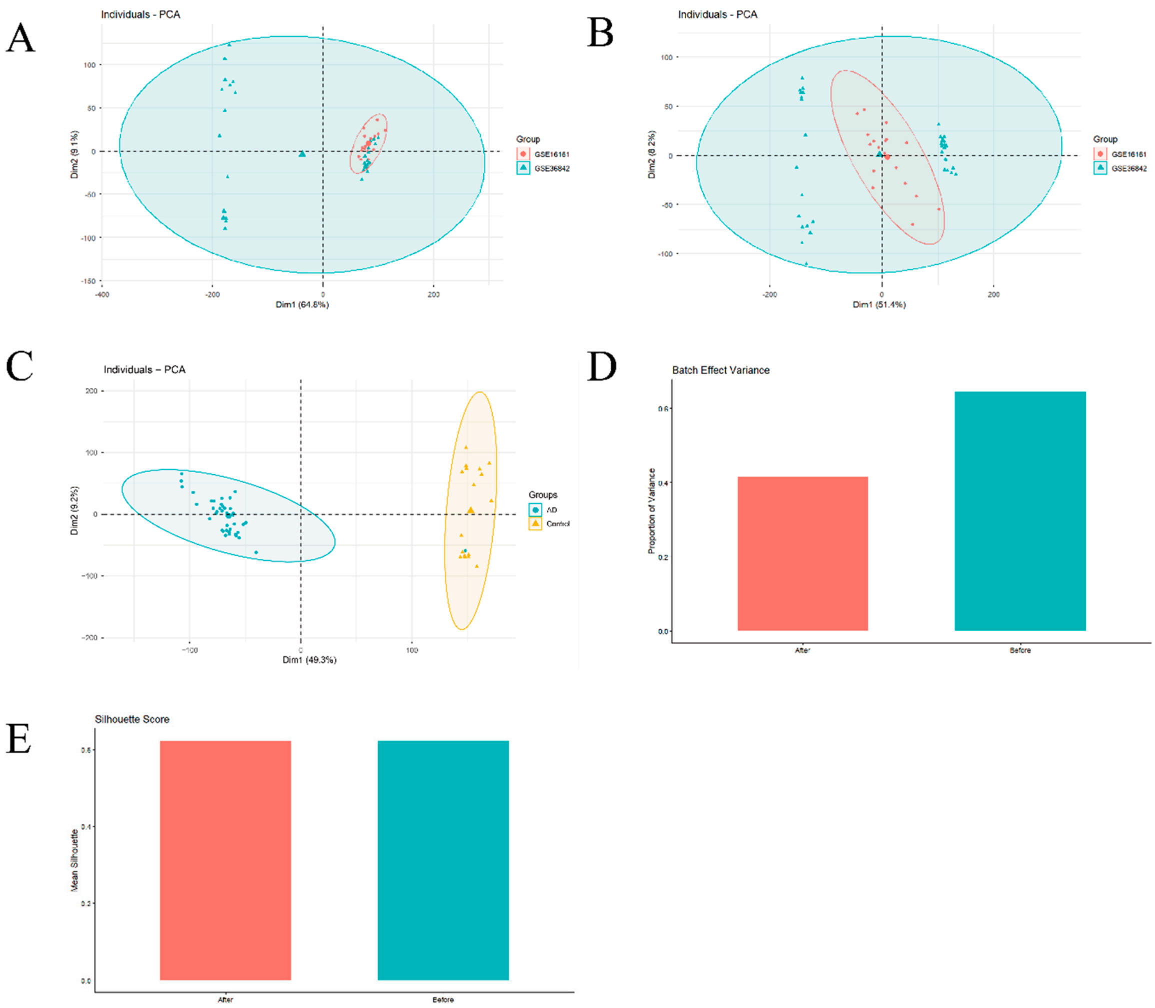

Figure S1 Effects of batch effect removal on AD datasets GSE36842 and GSE16161.

A. PCA analysis plot of GSE36842 and GSE16161 before batch effect removal. B. PCA component plot of GSE36842 and GSE16161 after batch effect removal in the AD datasets. C. PCA component plot of the AD group and control group in the AD datasets (GSE36842 and GSE16161) after batch effect removal. D. Reduction of batch-associated variance after ComBat correction based on variance partitioning analysis . E. Changes in sample clustering structure before and after batch correction evaluated by silhouette score

2. Ten-Fold Cross-Validation and Bootstrap Resampling Analysis of AUC Values in the AD Training Cohort

In the GSE6012 cohort (Figure 2A), the results of 10-fold cross-validation demonstrated that the C-index was close to 1.0 in most folds, with a significant decline (approximately 0.5) observed only in a few individual folds (e.g., Fold04). This indicates that the model exhibits high overall performance, yet there is a certain degree of fluctuation under some data partitions.

The corresponding bootstrap distribution (Figure 2B) revealed that most resampling results were concentrated in the high range (close to 1.0), while a certain degree of dispersion still existed. This suggests that the model performs well overall, but its stability is still influenced by the sample distribution.

In the GSE32924 cohort (Figure 2C), the 10-fold cross-validation results reached 1.0 in almost all folds, showing extremely high consistency. Further results from the bootstrap analysis (Figure

2D) indicated that the C-index distribution was highly concentrated around 1.0 with low dispersion, suggesting that the model exhibited strong stability on this dataset. In general, the results of the two validation methods consistently confirm that the model has good discriminative ability. However, it should be noted that the near-perfect performance (AUC/C-index ≈ 1.0) observed in some datasets may be associated with the small sample size and characteristics of the data distribution. Therefore, the relevant results should still be interpreted with caution.

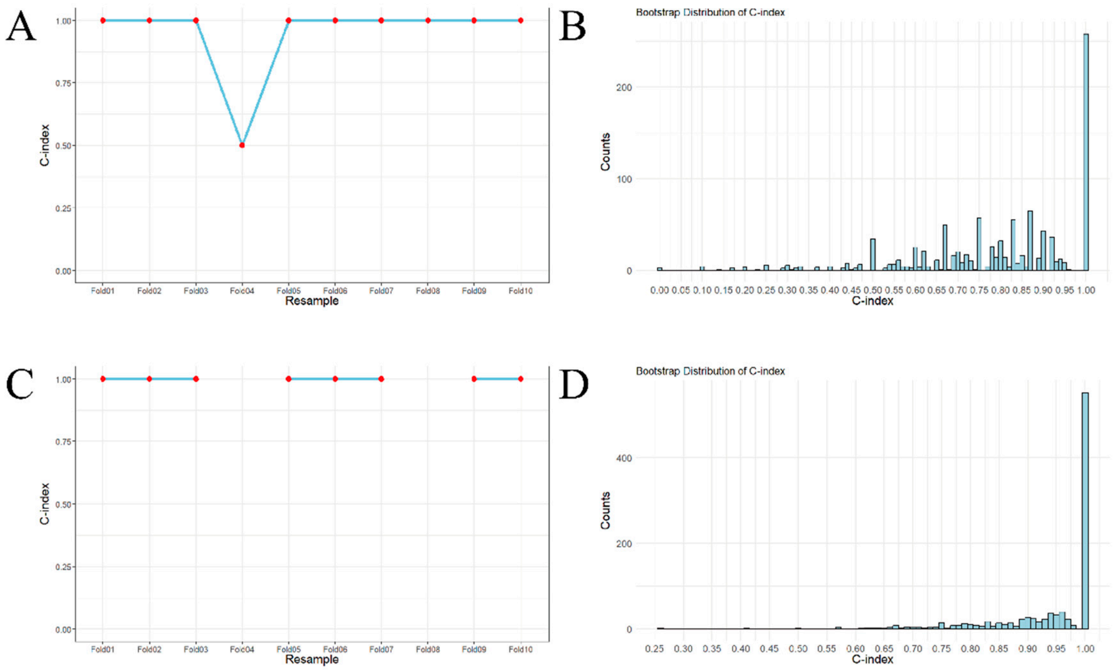

Figure S2 Ten-fold cross-validation of the reported AUC values in the atopic dermatitis validation cohort.
A. Ten-fold cross-validation in the GSE6012 cohort. B. Bootstrap resampling analysis in the GSE6012 cohort. C. Ten-fold cross-validation in the GSE32924 cohort. D. Bootstrap resampling analysis in the GSE32924 cohort.

3. Signature generated from machine learning based integrative approaches

In this study, machine learning methods were mainly applied in the stages of feature selection and prediction model construction. Specifically, on the basis of preliminary differential expression analysis and candidate gene screening, we adopted supervised learning methods (such as Lasso, Ridge, Enet, Stepglm, SVM, glmBoost, LDA, plsRglm, Random Forest, GBM, XGBoost, and Naive Bayes) for further screening and modeling of candidate features. These methods use the label information between disease and control groups to weight or select features, thereby identifying key biomarkers with strong discriminative ability.

Therefore, the machine learning methods adopted in this study belong to the supervised learning framework, and their core role is to screen the most predictive variables from candidate features and construct classification models, rather than being used for unsupervised clustering or pure statistical modeling.

Specifically, the multi-model construction strategy refers to the integrated modeling framework proposed in previous studies[1, 2]. Relevant studies have systematically evaluated the predictive performance of different algorithms in specific datasets by integrating multiple machine learning algorithms and constructing large-scale model combinations, thereby selecting models with good stability and generalization ability.

We drew on the "multi-algorithm combination + systematic evaluation" strategy in the aforementioned studies and constructed a total of 107 model combinations based on various machine learning methods. These models were not randomly generated, but derived from the systematic combination of different feature selection methods and classification/regression algorithms, covering various mainstream methods, including Lasso, Ridge, Elastic Net (Enet), Stepglm, Support Vector Machine (SVM), glmBoost, Linear Discriminant Analysis (LDA), plsRglm, Random Forest, GBM, XGBoost, and Naive Bayes classifier, etc. Under a unified data partitioning framework, we systematically trained and evaluated all models.

In terms of hyperparameter tuning, all models were optimized under the Leave-One-Out Cross-Validation (LOOCV) framework. Specifically, for Random Forest, grid search was performed on key parameters (such as the number of trees and the number of feature samples), and the optimal combination was selected based on C-index or related performance indicators; for Lasso, Ridge, and Elastic Net models, the regularization parameter λ was determined through LOOCV, where the α parameter of Elastic Net was traversed in the range of 0–1 with a step size of 0.1; for boosting models such as CoxBoost or glmBoost, the optimal penalty parameter was first determined using the corresponding function (such as optimCoxBoostPenalty), and then the optimal number of boosting steps was selected through cross-validation (such as cv.CoxBoost); in addition, the key parameters of models such as Stepglm, plsRglm, GBM, XGBoost, SVM, LDA, and Naive Bayes were also systematically optimized through cross-validation strategies, thereby ensuring that different models achieve optimal performance under a unified evaluation system. In the supplementary materials, we detailed all 107 model combination schemes and systematically presented the performance and comparison results of each combined model in different cohorts.

4. Expression of Hub Genes in the AD Validation Cohorts

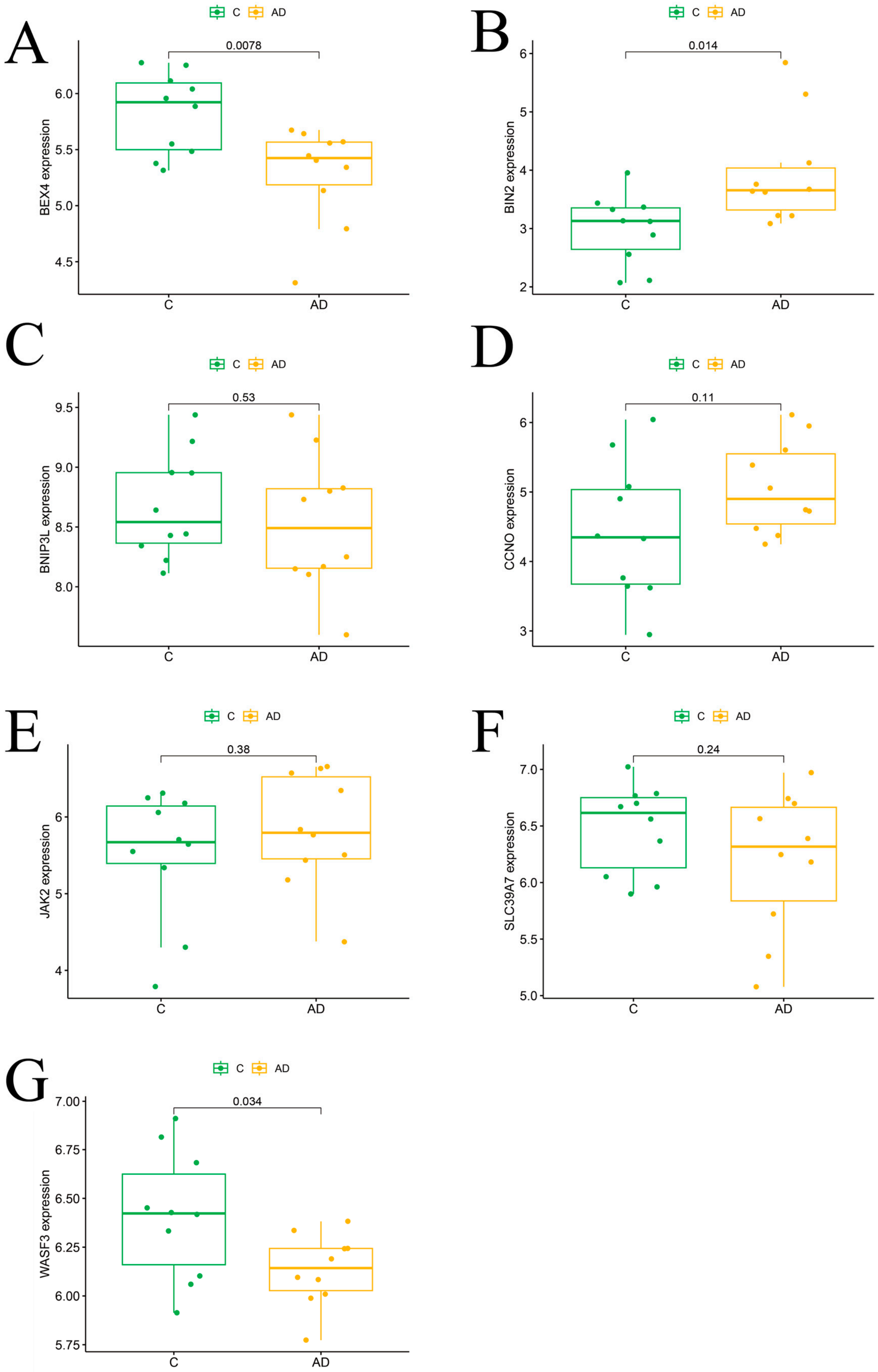

Figure S3. Expression levels of hub genes in the AD validation dataset GSE6012. A. Expression of BEX4 in AD and healthy control groups; B. Expression of BIN2 in AD and healthy control groups; C. Expression of BNIP3L in AD and healthy control groups; D. Expression of CCNO in AD and healthy control groups; E. Expression of JAK2 in AD and healthy control groups; F. Expression of SLC39A7 in AD and healthy control groups; G. Expression of WASF3 in AD and healthy control groups.

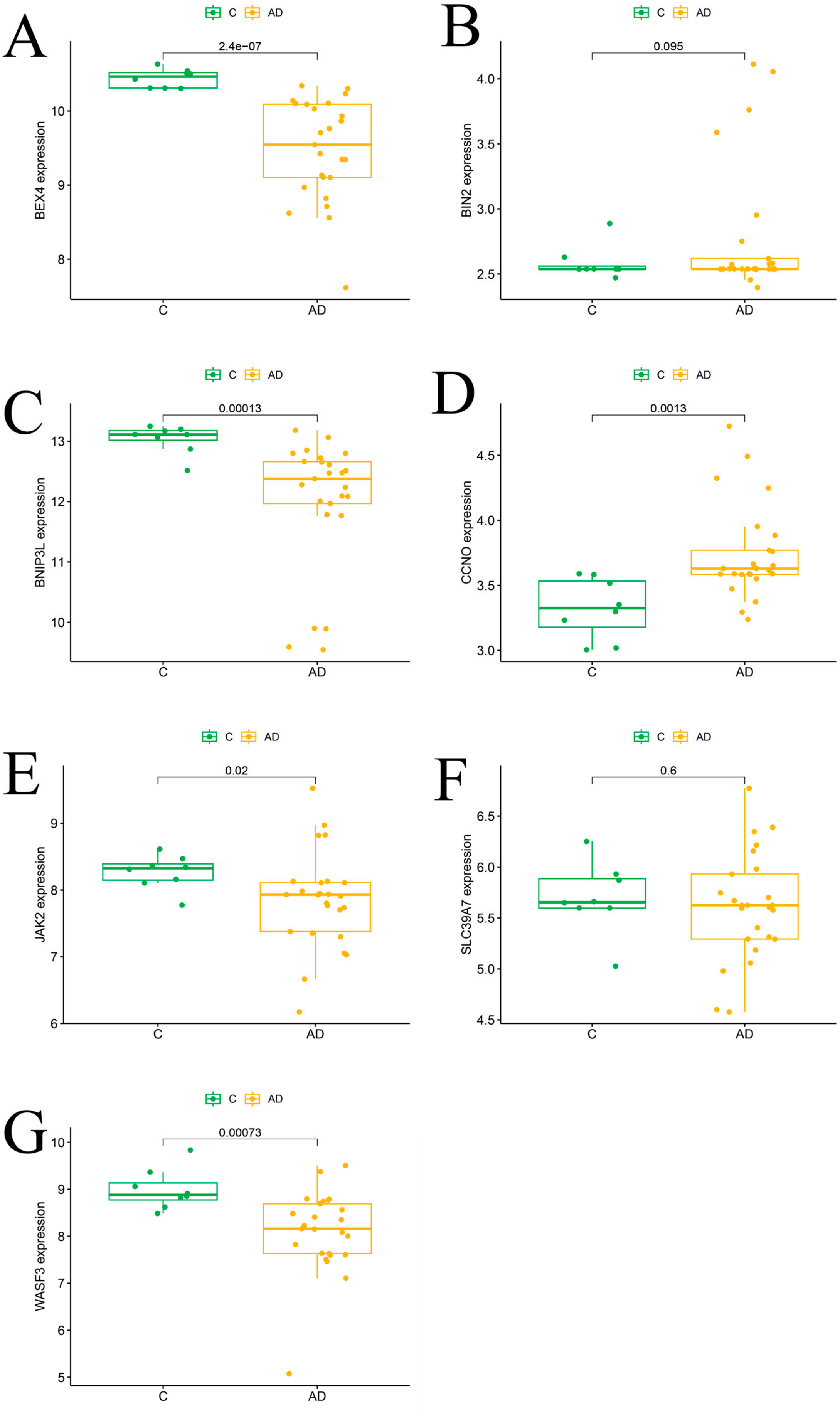

A
C
AD
2.4e−07
BEX4 expression
10
9
8
C
AD
B
C
AD
0.095
BIN2 expression
4.0
3.5
3.0
2.5
C
AD
C
C
AD
0.00013
BNIP3L expression
13
12
11
10
C
AD
D
C
AD
0.0013
CCNO expression
4.5
4.0
3.5
3.0
C
AD
E
C
AD
0.02
JAK2 expression
9
8
7
6
C
AD
F
C
AD
0.6
SLC39A7 expression
6.5
6.0
5.5
5.0
4.5
C
AD
G
C
AD
0.00073
WASF3 expression
10
9
8
7
6
5
C
AD

Figure S4. Expression levels of hub genes in the AD validation dataset GSE32924. A. Expression of BEX4 in AD and healthy control groups; B. Expression of BIN2 in AD and healthy control groups; C. Expression of BNIP3L in AD and healthy control groups; D. Expression of CCNO in AD and healthy control groups; E. Expression of JAK2 in AD and healthy control groups; F. Expression of SLC39A7 in AD and healthy control groups; G. Expression of WASF3 in AD and healthy control groups.

References:

[1]. Maimaiti, A., et al., DNA methylation regulator-mediated modification patterns and risk of intracranial aneurysm: a multi-omics and epigenome-wide association study integrating machine learning, Mendelian randomization, eQTL and mQTL data. J Transl Med, 2023. 21(1): p. 660.
[2]. Liu, Z., et al., Machine learning-based integration develops an immune-derived lncRNA signature for improving outcomes in colorectal cancer. Nat Commun, 2022. 13(1): p. 816.
